# Niacin Limitation Promotes *Candida glabrata* Adhesion to Abiotic Surfaces

**DOI:** 10.3390/pathogens11040387

**Published:** 2022-03-23

**Authors:** Xiaolan Li, Libang He, Bastiaan P. Krom, Lei Cheng, Johannes J. de Soet, Dong M. Deng

**Affiliations:** 1 Guanghua School of Stomatology, Guangdong Province Key Laboratory of Stomatology, Department of Operative Dentistry and Endodontics, Sun Yat-sen University, Guangzhou 510055, China; 2 Academic Centre for Dentistry Amsterdam (ACTA), Department of Preventive Dentistry, Universiteit van Amsterdam and Vrije Universiteit, 1081 LA Amsterdam, The Netherlands; b.krom@acta.nl (B.P.K.); jjdesoet@gmail.com (J.J.d.S.); d.deng@acta.nl (D.M.D.); 3 West China School of Stomatology, State Key Laboratory of Oral Diseases, National Clinical Research Center for Oral Diseases, Department of Operative Dentistry and Endodontics, Sichuan University, Chengdu 610041, China; helibang@scu.edu.cn (L.H.); chenglei@scu.edu.cn (L.C.)

**Keywords:** *Candida glabrata*, acrylic resin surface, hydrophobicity, adhesion gene, nicotinic acid

## Abstract

*Candida glabrata* is a prevalent fungal pathogen in humans, which is able to adhere to host cells and abiotic surfaces. Nicotinic acid (NA) limitation has been shown to promote the adherence of *C. glabrata* to human epithelial cells. Clinically, the elderly and hospitalized patients who are prone to *C. glabrata*–related denture stomatitis often suffer from vitamin deficiency. This study aimed to investigate *C. glabrata* adhesion to abiotic surfaces, including acrylic resin (a denture material) surfaces, cell surface hydrophobicity and adhesion gene expression. *C. glabrata* CBS138 was grown in media containing decreasing NA concentrations (40, 0.4, 0.04 and 0.004 µM). Adherence of *C. glabrata* to glass coverslips and acrylic resin was analyzed. *C. glabrata* adhesion to both surfaces generally increased with decreasing NA concentrations. The highest adhesion was found for the cells grown with 0.004 µM NA. The cell surface hydrophobicity test indicated that NA limitation enhanced hydrophobicity of *C. glabrata* cells. Quantitative PCR showed that of all adhesion genes tested, *EPA1*, *EPA3* and *EPA7* were significantly up-regulated in both 0.004 µM NA and 0.04 µM NA groups compared to those in the 40 µM NA group. No significant up- or down-regulation under NA limitation was observed for the other tested adhesion genes, namely *AWP3*, *AWP4*, *AWP6* and *EPA6*. NA limitation resulted in increased expression of some adhesion genes, higher surface hydrophobicity of *C. glabrata* and enhanced adhesion to abiotic surfaces. NA deficiency is likely a risk factor for *C. glabrata*–related denture stomatitis in the elderly.

## 1. Introduction

*Candida glabrata* is the second most prevalent fungal pathogen in humans after *Candida albicans*. For many years, *C. glabrata* was considered commensal in the normal flora of healthy humans. It is now considered one of the important pathogens in both mucosal and bloodstream infections [1]. It can rapidly disseminate throughout the body, causing the highest mortality among non-*albicans Candida* infections. Moreover, it is intrinsically more resistant to commonly used antifungal agents, such as azoles and echinocandins [1,2,3].

Clinically, *C. glabrata* bloodstream infections are commonly found in elderly individuals, diabetic patients and solid organ transplant recipients [4]. In the oral cavity, *C. glabrata* carriage and infection have been associated with denture wearing, hospitalization and increasing age [5,6,7]. In the elderly, the increase of *C. glabrata* appeared to be the most significant among all *Candida* species [4,7,8]. It is known that aging generally leads to increased colonization by bacteria and fungi, due to physiological changes. for example, a decreased flow of saliva in the elderly [9]. However, at the moment, it is unclear why the increase of *C. glabrata* is particularly observed in the elderly.

An important virulence factor of *C. glabrata* is its capacity to strongly adhere to host cells and surfaces of various medical devices [10,11,12,13]. The genome of *C. glabrata* contains an exceptionally large number (67 in the CBS138 genome) of adhesin-like genes [14]. These are classified into several subclasses based on their N-terminal substrate-binding domain. Among these subclasses, both Epa (Epithelial adhesion) proteins and Awps (adhesin-like cell wall) proteins have been shown to mediate adhesion and biofilm formation of *C. glabrata* and are important for its pathogenicity [10]. The family of Epa adhesins have been studied most frequently. These proteins have been shown to facilitate the binding of *C. glabrata* to host cells through ligand recognition, although most proteins possess individual tailored ligand binding properties [11]. These functionally diverse lectin-like adhesins of *C. glabrata* might allow it to recognize various types of host cell carbohydrates and to facilitate efficient host invasion and dissemination [13].

*C. glabrata* is also able to adhere to abiotic surfaces, including the surfaces of urinary catheters, prosthetic valves [12] and dentures. Several in vitro studies showed that *C. glabrata* exhibited a stronger capacity to adhere to denture acrylic surfaces than other microorganisms [15,16]. In vivo, the number of yeast cells in biofilms formed on acrylic resin was significantly higher for *C. glabrata* compared to other *Candida* species 7 days after wearing the denture [17]. The genetic mechanism of the adherence of *C. glabrata* to abiotic surfaces was recently studied. It was demonstrated that disruption of sub-telomeric silencing could dramatically enhance the adhesion of *C. glabrata* to hydrophilic and hydrophobic surfaces via de-repression of Epa proteins, particularly Epa1, Epa6 and Epa7 [18]. Hence, it was proposed that Epas are multi-modal adhesins. They function not only via specific lectin-glycan interactions but also via nonspecific hydrophobic or hydrophilic interactions mediated by their large central glycosylated domains [19].

Domergue et al. [20] reported that one of the essential vitamins, B3 (nicotinic acid, niacin, NA), affected the adherence of *C. glabrata* in urinary tract infections. NA is a precursor of nicotinamide adenine dinucleotide (NAD+). *C. glabrata* is an NA auxotroph microorganism and depends on an environmental supply of vitamin precursors of NAD+ for growth. Limitation of NA in urine de-repressed adhesin genes from subtelomeric silencing, leading to enhanced adhesion of *C. glabrata* to uroepithelial cells in vitro and to the bladder-wall in a mouse urinary tract infection model, through the upregulation of *EPA1*, *EPA6* and *EPA7*. Since these three Epa proteins mediated the adherence of *C. glabrata* to abiotic surfaces, we hypothesize that the NA limitation could enhance the adherence of this *Candida* species to abiotic surfaces through up-regulation of adhesion genes as well.

The aim of this study was to investigate the effect of NA limitation on *C. glabrata* adhesion to abiotic hard surfaces, including acrylic resin surfaces, cell surface hydrophobicity and adhesion gene expression.

## 2. Results

### 2.1. C. glabrata Growth under Various NA Concentrations

The growth of *C. glabrata* in a chemically defined medium (CDM), supplemented with various NA concentrations was first examined. Growth curves were used to determine the range of NA concentrations and the duration for a *C. glabrata* planktonic culture to reach stationary phase growth.

*C. glabrata* is a NA auxotroph, which requires NA to grow. In the pilot study, we found that *C. glabrata* was not able to grow when the NA concentration was lower than 0.004 µM. Hence, the lowest NA concentration selected for this study was 0.004 µM. The NA concentration of 0.4 µM was chosen to mimic salivary NA concentrations in healthy individuals, which is reported to be around 0.25 µM [21,22]. The original CDM used for the growth of *C. glabrata* in our laboratory contains 40 µM NA (excess NA). Therefore, the range between 0.004 µM and 40 µM was chosen as the NA test range in this study.

**Figure 1:**
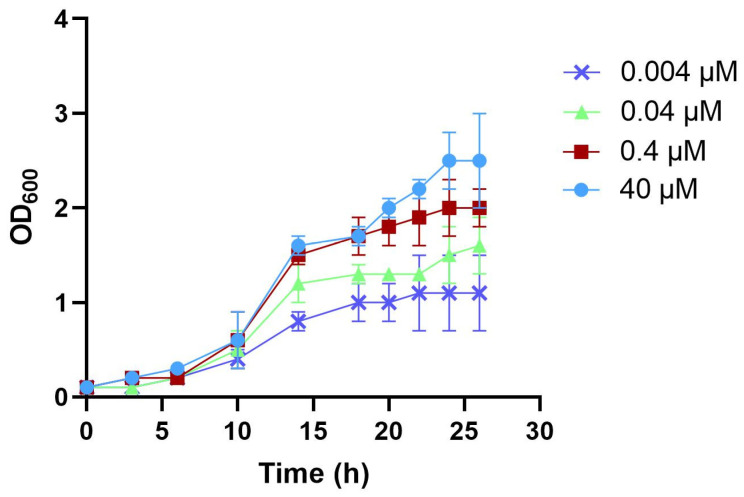
Growth curves of *C. glabrata*. The *C. glabrata* cells were grown in chemically defined medium (CDM) supplemented with 40, 0.4, 0.04 and 0.004 µM NA. The planktonic cultures were grown aerobically, shaking at 150 rpm, at 30 °C. The OD_600_ value of the cell culture was recorded in time. The growth experiment was repeated three times. Each point represents the mean ± standard deviation (SD).

Figure 1 shows that the NA concentration clearly affected the growth rate and the final OD values of the stationary cultures of *C. glabrata*. A previous study showed that stationary grown cells adhered better to a surface and expressed more adhesin-like wall proteins than exponentially growing cells [14]. Therefore, stationary grown cultures, instead of exponential cultures, were chosen for adherence and hydrophobicity tests. Based on the growth curves, we chose 22 h as the time point of cell collection for the subsequent tests. At 22 h, all cultures reached early stationary growth phase.

### 2.2. Adherence of C. glabrata to Abiotic Surfaces

**Figure 2:**
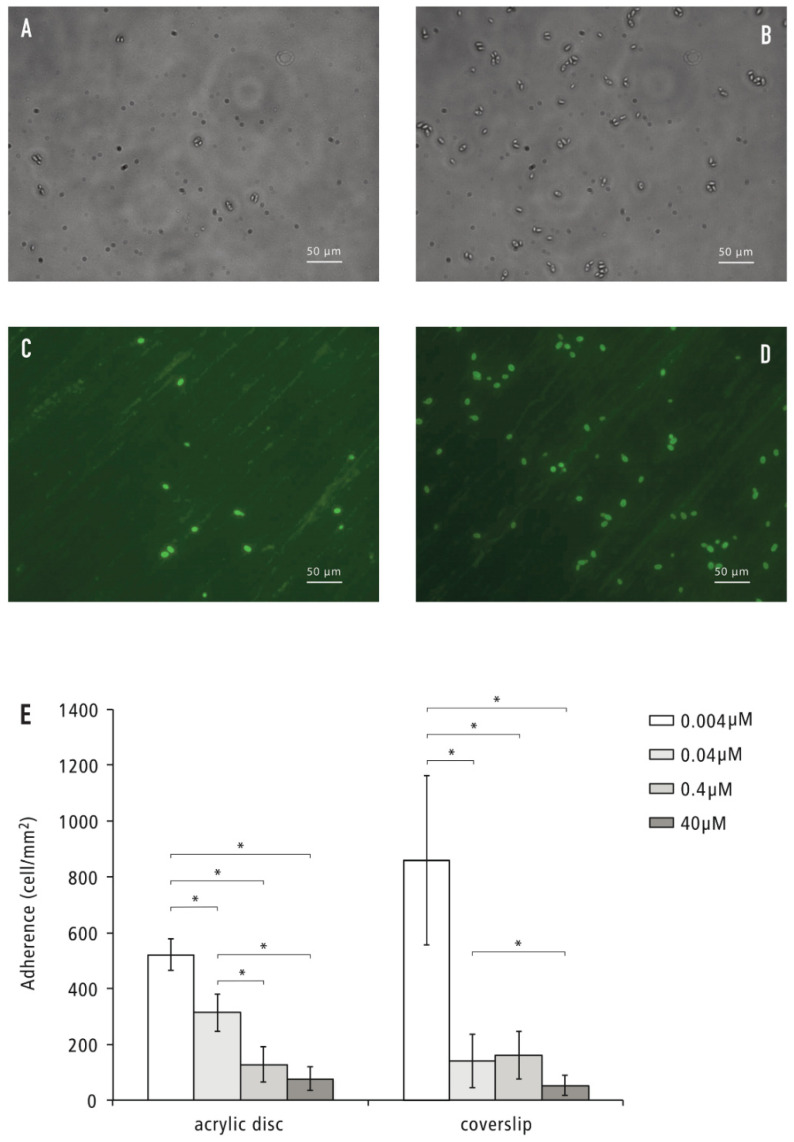
Adherence of *C. glabrata* to acrylic resin discs and glass coverslips. *C. glabrata* cells were suspended in PBS (OD_600_ = 0.25) and incubated with the substrata for 2 h under shaking conditions. Images were taken under 40× magnification. The experiment was repeated three times for glass coverslips and two times for acrylic resin. In each experiment, three replicates were used per NA concentration per substratum type. (**A**–**D**): Representative microscopy images of adhered *C. glabrata* cells. (**A**) NA 40 µM, glass coverslip surface; (**B**) NA 0.004 µM, glass coverslip surface; (**C**) NA 40 µM, acrylic resin surface; (**D**) NA 0.004 µM, acrylic resin surface; (**E**) Number of adhered cells per mm^2^ of substrata surface. The number of adhered cells (presented as cells per mm^2^) was determined from nine randomly selected images of each sample. The cell number per experiment was calculated by averaging the cell numbers of three replicate samples. The data presented are the average adhered cell numbers of two to three experiments. Each bar represents the mean ± standard deviation (SD). * Indicates statistically significant difference between two groups (*p* < 0.05). The size bar indicates 50 µm.

*C. glabrata* cells grown under various NA concentrations were tested for their ability to adhere to glass coverslips or acrylic discs. Two substratum types were used, because a previous study showed that the properties of the type of substratum affects the adhesion behavior of *Candida* species [23]. Figure 2A,B shows representative bright-field images of *C. glabrata* adhesion to glass coverslips. Figure 2C,D shows representative fluorescent images of *C. glabrata* adhesion to acrylic resin. Figure 2E presents the quantified data. Bright-field images were taken because the adhered *C. glabrata* on glass coverslips could be observed directly under a light microscope. The adhered cells on acrylic discs were stained with Concanavalin A and Alexa Fluor^®^ 488 Conjugate and observed under a fluorescent microscope because of the opaque characteristics of acrylic resin discs.

For acrylic resin, the adhesion of *C. glabrata* significantly increased with decreasing NA concentration in the growth media, whereas for the glass coverslip, this NA concentration-dependent adhesion was less pronounced. Clearly, the NA-dependent adhesion properties of *C. glabrata* were different between glass coverslips and denture material acrylic resin, even though both materials are abiotic. Despite the observed different adhesion pattern, the NA concentration of 0.004 µM significantly enhanced adherence of *C. glabrata* to both substrata (*p* < 0.05).

### 2.3. Cell Surface Hydrophobicity

**Figure 3:**
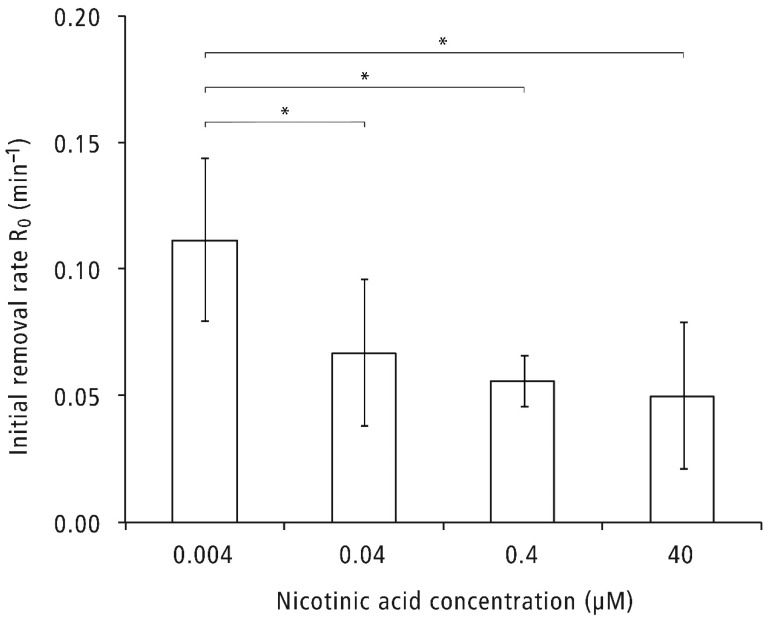
Hydrophobicity of *C. glabrata* cells. *C. glabrata* cells were suspended in PBS (OD_600_ = 0.4–0.6) and subjected to a kinetic MATH test. The experiment was repeated three times. In each experiment, the measurements were repeated four to five times per NA concentration. The R_0_ value per NA concentration per experiment was the average of the repeated measurements. The data presented are the average R_0_ values of three experiments. Each bar represents the mean ± standard deviation (SD). * Indicates statistically significant difference between two groups (*p* < 0.05).

A kinetic MATH test was used to determine the cell surface hydrophobicity of *C. glabrata* [24]. Higher initial removal rates (R_0_) indicate higher cell surface hydrophobicity in a kinetic MATH test, provided the assay conditions, most notably the buffer composition, are kept constant. In this study, the stationary *C. glabrata* cells were collected by centrifugation and resuspended in PBS buffer before the kinetic MATH test. Figure 3 shows the R_0_ of *C. glabrata* cells grown under various NA concentrations. The R_0_ of the culture grown with the lowest NA concentration (0.004 µM) was the highest among all tested groups, whereas the R_0_ of other NA concentration groups was similar and significantly lower than the NA 0.004 µM group.

### 2.4. Adhesion Gene Expression

**Figure 4:**
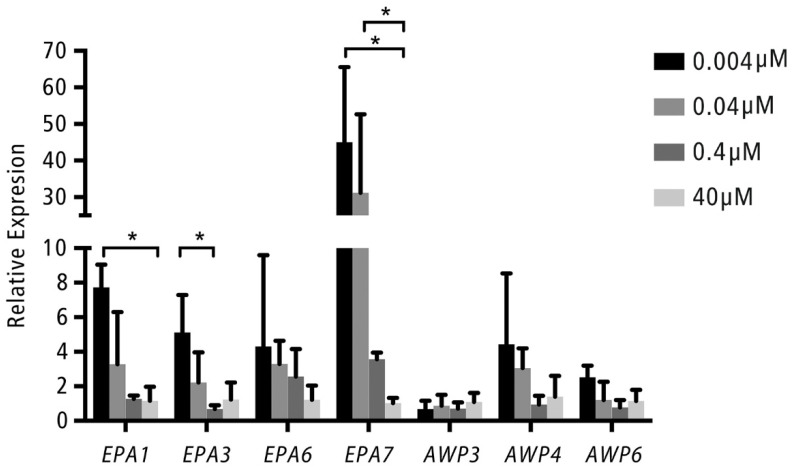
Relative gene expression of several adhesion genes. The relative expression of each selected gene in *C. glabrata* CBS138 grown in the medium containing 0.004, 0.04, and 0.4 µM NA relative to those grown in 40 µM NA. Each bar represents the mean ± standard deviation (SD). * Refers to statistically significant up-regulation (*p* < 0.05). The experiment was repeated three times. In each experiment, three replicates were used per NA concentration.

Of the known adhesion genes (*EPA1*, *EPA6* and *EPA7*) and the reported biofilm-related adhesion genes (*EPA3*, *AWP3*, *AWP4*, *AWP6*), the expression of *EPA1* and *EPA7* in the cultures of the 0.004 µM NA group was significantly up-regulated seven-fold (for *EPA1*) to 45-fold (for *EPA7*), as compared to those in the cultures of the 40 µM NA group. The expression of *EPA7* in the 0.04 µM NA group was also 31-fold higher than that in the 40 µM NA group. In the case of *EPA3*, its expression in the 0.004 µM NA group was significantly higher than in the 0.4 µM NA group and marginally higher than in the 40 µM NA group (*p* = 0.08). No significant up- or down-regulation was observed for *AWP3*, *AWP4*, *AWP6* and *EPA6* genes (Figure 4).

## 3. Discussion

In this study, we examined the influence of NA limitations on the adherence of *C. glabrata* cells to hard/abiotic surfaces and explored the underlying mechanism by measuring cell surface hydrophobicity and expression of adhesion genes. Our data demonstrated that NA limitation induced the expression of the adhesion genes *EPA1*, *EPA3* and *EPA7*, which might consequently increase the surface hydrophobicity of *C. glabrata* and enhance its adhesion to glass and acrylic resin surfaces.

A previous study showed that NA limitation induced *C. glabrata* adherence to biotic surfaces, uroepithelial cells and bladder wall [20]. Our study broadened this finding by showing that NA limitation could induce *C. glabrata* adhesion to abiotic glass or acrylic resin surfaces as well. In healthy individuals, the NA concentration in saliva was estimated to be 0.25 µM [21,22]. In this study, the NA concentration of 0.4 µM mimicked a healthy salivary environment. No clear difference in *C. glabrata* adhesion was seen between cells grown under 0.4 µM NA and excess NA (40 µM). However, significantly higher adhesion to acrylic resin surface was observed when the NA concentration was lowered 10-fold. The risk of *C. glabrata* infection in denture wearers would likely increase when salivary NA concentrations were reduced 10-fold. Further clinical investigation is needed to establish this link between NA limitation and *C. glabrata* prevalence or infection in denture stomatitis patients.

The study of Domergue et al. suggested that NA limitation could reduce the activity of the NAD+ -dependent histone deacetylase Sir2p, leading to the loss of silencing of multiple *EPA* genes, which are regulated by sub-telomeric silencing. Three telomeric *EPA* genes, *EPA1*, *EPA6* and *EPA7*, were induced by NA limitation [20]. However, in our study, although there is a trend that the expression of *EPA6* increased with the decreasing NA concentrations, this increase did not reach statistical significance. The different findings on *EPA6* expression between the two studies could be related to the different growth media used in both studies. The medium in the previous study was designed to mimic urine (pH 4.0), whereas the medium used in the current study represents the resting pH of saliva (pH 7.0). Using RNA-seq, Linde et al. [25] revealed that *EPA6* expressed different isoforms at pH shift. One of the isoforms was about five-fold up-regulated in pH 4 as compared to pH 8. This evidence might provide an explanation of the discrepancy between the results of our study and Domergue’s studies.

Another difference between our study and Domergue’s study is the *C. glabrata* strain tested. We used the strain ATCC2001 (CBS138), whereas Domergue’s study used strain BG2. It is known there is huge variability in the number and type of adhesin-coding genes in different strains of *C. glabrata* isolated from patients worldwide [26]. In terms of the regulation of adhesin gene expression, so far, the most information on sub-telomeric silencing has been obtained from strain BG2 [19,20,27,28]. Halliwell et al. [29] discovered that Sir-dependent transcriptional silencing was the primary mechanism in regulating EPA1 expression in strain BG2 but not in CBS138. Under NA-limitation (NA 0.167 µM), the expression of EPA1 was upregulated in BG2, whereas it was unaffected (or decreased) in CBS138. This finding seemed to disagree with what was reported in the current study, where a significant upregulation of *EPA1* expression was observed under NA limitation in the same CBS138 strain. When comparing the NA concentrations tested in the two studies, we noticed that the concentration of NA in our study, 0.4 µM, was comparable to the level of NA limitation in Halliwell’s study. Both studies did not observe any changes in *EPA1* expression. Halliwell et al. [29] showed that EPA1 silencing is weakened but not blocked completely in strain CBS138, and the presence of an additional copy of the *SIR3* gene helped establish the silencing. Moreover, the *SIR3* mRNA levels were approximately 1.5-fold higher in CBS138 than in BG2. Hence, our results seem to indicate that strain CBS138 is less sensitive to NA deficiency than strain BG2. This will be an interesting topic for further research, but is not within the scope of the present study.

In this study, we examined the expression of genes from both Epa and Awp families in *C. glabrata* grown under various NA concentrations. Three *AWP* genes were examined, because upregulation of these genes in biofilms has been reported [30,31,32]. It seems that the expression of the tested *AWP* genes was unaffected by the NA concentrations. As for the Epa family, in line with the previous finding, which demonstrated that NA limitation induced the upregulation of *EPA1* and *EPA7* in the BG2 strain [20], our results showed that a similar phenomenon was observed in the CBS138 strain. In addition, we found that the expression of *EPA3* was also induced by NA limitation. Previously, the roles of *EPA1* and *EPA7* in the adhesion of *Candida* to abiotic surfaces have been proven by using knockout strains for these genes [19]. The absence of these two genes reduced the adhesion of *Candida*. However, it should be noted that these knockout strains were derived from the BG2 strain. Given the strain differences mentioned above, it is worthwhile to construct the null mutant strain from strain CBS138 and to investigate the involvement of *EPA1*, *EPA3* and *EPA7* in the adherence of *C. glabrata* CBS138 to abiotic surfaces with these mutant strains.

It has been suggested that cell surface hydrophobicity could play a role in *C. glabrata* adhesion [5]. Therefore, we examined the cell surface hydrophobicity of *C. glabrata* grown under four NA concentrations. At the lowest NA concentration, the hydrophobicity was significantly higher than at other NA concentrations, which coincided with the *C. glabrata* adhesion pattern on glass coverslips. However, the cell wall hydrophobicity alone could not explain the NA-dependent adhesion to acrylic resin. It is known that the properties of the substrata surfaces could affect the adhesion behavior of *Candida* species [23]. In our study, the surface wettability and surface roughness of the tested substrata were clearly different; hence, it is possible that surface properties of the substrata played a role in the different *Candida* adhesion patterns on glass coverslips and acrylic resins. To further understand how NA limitation influences the interaction between *C. glabrata* and a substratum, a follow-up study using the single-cell atomic force microscopy in combination with the adhesin gene mutants based on the relevant genes identified from this study could be useful.

## 4. Materials and Methods

### 4.1. Strain and Growth Medium

*C. glabrata* ATCC2001/CBS138 (Centraalbureau voor Schimmelcultures, Utrecht, the Netherlands) was aerobically grown at 30 °C on YPD agar (1% yeast extract, 2% peptone, 2% glucose, 1.5% agar) or in a chemically defined medium (CDM) while shaking at 150 rpm. CDM contained 18 mM glucose, 76 mM K_2_HPO_4_, 15 mM KH_2_PO_4_, 10 mM (NH_4_)_2_SO_4_, 35 mM NaCl and 2 mM MgSO_4_•7H_2_O, supplemented with filter-sterilized vitamins (0.1 mM pyridoxine–HCl, 0.01 mM pantothenic acid, 1 µM riboflavin, 0.3 µM thiamine-HCl and 0.05 µM d-biotin) and amino acids (4 mM L-glutamic acid, 1 mM L-arginine HCl, 1.3 mM L-cysteine HCl, and 0.1 mM L-tryptophan). Various volumes of the filter-sterilized NA stock were added to the medium in order to achieve the final NA concentrations of 40, 0.4, 0.04 and 0.004 µM. Since *C. glabrata* is an NA auxotroph, a minimum of NA 0.004 µM is required for its growth, as has been tested in pilot experiments. The final pH of the medium was adjusted to 7.0 with KOH.

### 4.2. Substrata

Two types of substrata were used to examine the adhesion of *C. glabrata*: glass coverslips (Thermo Scientific, Braunshweig, Germany) and acrylic resin discs, both with diameters of 10 mm. Acrylic resins are used for the base of dentures. This study used acrylic resin discs to mimic oral dentures. These discs were prepared according to the general procedure of denture preparation. In detail, a heat-polymerized acrylic resin (Dentsply International, York, PA, USA) and its monomer (Dentsply International) were mixed, packed into a disc-shaped mold (10 mm in diameter and 1.0 mm in height) and polymerized for 10 h at 80 °C, following by cooling at room temperature for 2 h. The polymerized acrylic resin discs were then immersed in distilled water for 14 h at 37 °C to release the residual monomer. Before use, the resulting discs were polished using silicon carbide papers (400, 600 and 1200 grit) and ultrasonically cleaned in an ultrasonic cleaning unit (Easyclean, Renfert, Hilzingen, Germany) for 30 min. The surface wettability of these two substrata types was evaluated by measuring the static contact angles with drops of distilled water. The contact angle of the coverslips and the heat-polymerized acrylic resin was 42.77° ± 0.79° and 61.25° ± 2.55°, respectively. The surface roughness (Ra) of the coverslips and the heat-polymerized acrylic resin was 2.83 ± 1.6 nm and 68.17 ± 25.5 nm, respectively.

### 4.3. Growth under Nicotinic Acid Limitation

A pre-culture was obtained by growing *C. glabrata* in CDM supplemented with 40 µM NA (excess NA) for 24 h. Cells were harvested by centrifugation (3939× *g*, 5 min), washed twice in CDM without NA and resuspended in CDM containing 40, 0.4, 0.04 and 0.004 µM NA to a final optical density of 0.1, measured at 600 nm (OD_600_). Cultures were further grown until the cells reached stationary phase (22 h). For the adherence and hydrophobicity tests, the stationary grown cells were harvested by centrifugation (3939× *g*, 5 min) and resuspended in phosphate-buffered saline (PBS; NaCl 8 g, KCl 0.2 g, Na_2_HPO_4_ 1 g, KH_2_PO_4_ 0.2 g, per liter, pH 7.4) to the desired OD: OD_600_ = 0.25 for adherence tests and OD_600_ = 0.4–0.6 for hydrophobicity tests. The stationary grown cultures were also processed for total RNA extraction, followed by adhesion gene expression analysis.

### 4.4. Adherence Assay

**Figure 5:**
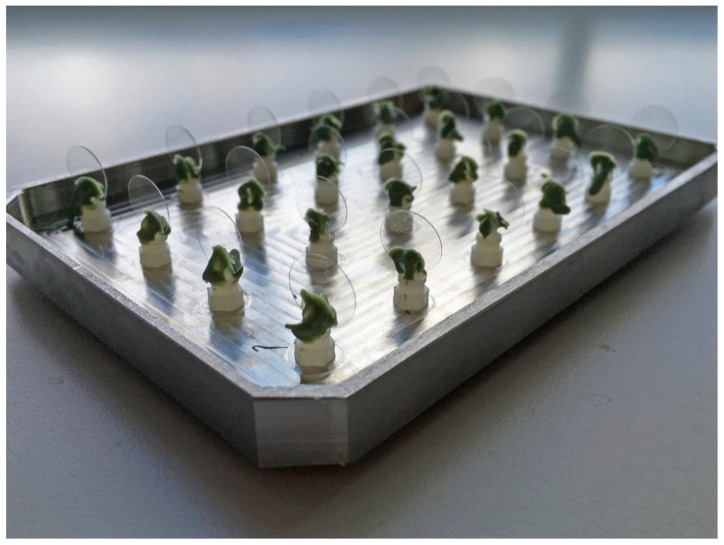
An image of the active attachment model. In this model, a custom-made stainless-steel lid was fixed with 24 clamps. The substrata, acrylic resin discs or glass coverslips (shown in the image), were inserted in the clamps. For the adherence assay, the metal lid with substrata was placed into a 24-well plate containing *C. glabrata* suspensions [33].

Both types of substrata were installed on a custom-made metal lid of an active attachment model (Figure 5). The lid was sterilized either by autoclaving (glass coverslips) or by UV light (acrylic resin discs). The *C. glabrata* suspensions (1.5 mL, OD_600_ = 0.25) were placed into wells of a 24-well plate (NuncTM, Roskilde, Denmark). Subsequently, the metal lid with substrata was placed into the 24-well plate and was then incubated at 37 °C with shaking (35 rpm) for 2 h. The duration of 2 h was chosen to obtain measurable adherence but without obvious growth of *C. glabrata*. After 2 h, the substrata were washed twice in PBS (1.6 mL/well) and fixed in 3.7% formaldehyde for 2 h at room temperature. The coverslips were mounted on a glass slide, and the adhesion of *C. glabrata* was quantified by microscopic analysis (Carl Zeiss Axioskop) using a 40× objective. The acrylic resin discs were stained with 50 μg/mL of Concanavalin A and Alexa Fluor^®^ 488 Conjugate (Life technologies, Bleiswijk, The Netherlands) in the dark for 45 min. Adhesion was quantified using a fluorescence microscope (EVOS^®^ FL cell imaging system, Life technologies) with a 40× objective using the appropriate filter settings. The number of adhered cells (presented as cells per mm^2^) was manually counted from 9 randomly selected images of each sample. The experiment was repeated 3 times for glass coverslips and 2 times for acrylic resin. In each experiment, 3 replicates were used per NA concentration per substratum type. The cell number per experiment was calculated by averaging the cell numbers of 3 replicate samples. The results are presented as the average cell numbers of 2 (acrylic resin) or 3 (glass coverslips) experiments.

### 4.5. Microbial Adhesion to Hexadecane (MATH)

*C. glabrata* cells were resuspended in PBS (ionic strength 0.15 M, pH 7.4). The resuspensions were adjusted to an OD_600_ of 0.4–0.6 (A_0_), and 150 µL of hexadecane was then added to 3 mL cell suspension in a polystyrene macro cuvette (BRAND^®^, Wertheim, Germany). The two-phase system was vortexed (Fisher Scientific Vortex Shaker, 40 Hz, Hanover Park, IL, USA) for 10 s and allowed to settle for 10 min before the OD_600_ was measured (A_t_). This was repeated for 4–5 times, and log (A_t_/A_0_ × 100) was plotted against time. Linear least-square fitting subsequently yielded the initial removal rate R_0_ (min^−1^), which is a measure of the cell surface hydrophobicity. The experiment was repeated 3 times. In each experiment, the measurements were repeated 4–5 times per NA concentration. The R_0_ value per NA concentration per experiment was the average of the repeated measurements. The data presented are the average R_0_ values of three experiments.

### 4.6. Adhesion Gene Expression by Quantitative PCR

*C. glabrata* stationary culture (22 h) was centrifuged, and the cell pellets were immediately resuspended in 1 mL RNA-protect Bacteria Reagent (Qiagen GmbH, Hilden, Germany) and stored at −80 °C until analysis.

Total RNA was extracted by beating with 0.5 mm glass beads followed by RNA purification using the Qiagen RNeasy Mini Kit (Qiagen GmbH, Hilden, Germany) according to the manufacturer’s instructions. All RNA extraction steps were performed on ice. Genomic DNA contamination was removed with the TURBO DNA-*free*^TM^ Kit (Life Technologies Carlsbad, CA, USA). Subsequently, cDNA was synthesized using a First Strand cDNA Synthesis Kit (Thermo scientific, Waltham, MA, USA) with both Oligo(dT)18 and random hexamer D(N)6 primers. The expression of adhesion genes, *EPA1*, *EPA3*, *EPA6*, *EPA7*, *AWP3*, *AWP4* and *AWP6*, was examined with gene-specific PCR primers, using SYBR^®^ Green based quantitative PCR (qPCR). Primer sequences and annealing temperatures are given in Table 1 [30,34]. The specificity of PCR reactions was confirmed by melting curve analysis. Expression levels were normalized using the expression of 2 housekeeping genes, 18S rRNA and *PGK1* (Phosphoglycerate kinase). The relative quantification of gene expression was calculated by the 2(−ΔΔC(_T_)) method [35].

### 4.7. Statistical Analysis

Data was analyzed with the Statistical Package for Social Science (Version 17.0, SPSS, Chicago, IL, USA). A one-way analysis of variance (ANOVA) was used to evaluate the effect of NA concentrations on the number of adhered cells, hydrophobicity and gene expression. The post hoc comparisons were carried out with Bonferroni correction for multiple testing. *p* < 0.05 was considered as statistically significant.

## 5. Conclusions

In summary, combined with the evidence from the previous study [20], we can conclude that NA limitation promoted the adherence of *C. glabrata* to both epithelial cells and abiotic materials. For a clinical situation, this finding indicates that patients with NA deficiency could be prone to *C. glabrata* colonization and *C. glabrata*–related denture stomatitis. Since elderly people and hospitalized patients are often deficient in key vitamins [36] and are prone to *C. glabrata* infection, our study may provide scientific grounds for new prevention and treatment strategies against *C. glabrata* oral infection in the elderly by supplementing vitamin B3 in these patients.

## Figures and Tables

**Table 1:** Primer sequences and reaction temperatures.

Gene		Primer Sequences (5′–3′)	Annealing Temperature (°C)
*EPA1*	F	TTCAGACCAAAAGTAACTGGCTTC	57
	R	CCTAATAGGGTAATATACGCCCG	
*EPA3*	F	TGGATGTTCTCCTCAGGATGTTG	55
	R	TGTAGACCAGTTGTTTGAGCCTTG	
*EPA6*	F	TGATTATTTGAAATCAGGATCGAATC	55
	R	TGTCATTGTCAATGGTGTACGATAG	
*EPA7*	F	GATTTACGGAAGAATGGTTCGTAC	55
	R	GGTAAATGATCTATTTCGGGTGTG	
*AWP3*	F	GCCCAGATCAATGGAGCGG	57
	R	CACAGCGATTGACGTAACACCAG	
*AWP4*	F	CAATTACGATGTCCTGGATAATCCGR	59
	R	CTTGAAAGGCTAAGTAAACACCTCC	
*AWP6*	F	CGCATTGGCTGCGGTAGC	59
	R	TGGCCTTTGATATCAGCCAAG	
*18S rRNA*	F	CCGAGGACTGCGATACTTGT	57
	R	CACCCAAACACTCGCATAGA	
*PGK1*	F	ACGAAGTTGTCAAGTCCTCCA	57
	R	TTACCTTCCAACAATTCCAAGGAG	

Abbreviations: F forward; R reverse.

## Data Availability

Raw data can be requested by contacting the corresponding authors.
